# Establishing a cost-per-result of laboratory-based, reflex Cryptococcal antigenaemia screening (CrAg) in HIV+ patients with CD4 counts less than 100 cells/μl using a Lateral Flow Assay (LFA) at a typical busy CD4 laboratory in South Africa

**DOI:** 10.1371/journal.pone.0171675

**Published:** 2017-02-06

**Authors:** Naseem Cassim, Kathryn Schnippel, Lindi Marie Coetzee, Deborah Kim Glencross

**Affiliations:** 1 National Health Laboratory Service (NHLS), National Priority Programmes, Johannesburg, South Africa; 2 Department of Molecular Medicine and Haematology, Faculty of Health Sciences, University of Witwatersrand, Johannesburg, South Africa; 3 Right to Care, Johannesburg, South Africa; Central University of Tamil Nadu, INDIA

## Abstract

**Introduction:**

Cryptococcal meningitis is a major cause of mortality and morbidity in countries with high HIV prevalence, primarily affecting patients whose CD4 are < = 100 cells/μl. Routine Cryptococcal Antigen (CrAg) screening is thus recommended in the South African HIV treatment guidelines for all patients with CD4 counts < = 100 cells/μl, followed by pre-emptive anti-fungal therapy where CrAg results are positive. A laboratory-based reflexed CrAg screening approach, using a Lateral Flow Assay (LFA) on remnant EDTA CD4 blood samples, was piloted at three CD4 laboratories.

**Objectives:**

This study aimed to assess the cost-per-result of laboratory-based reflexed CrAg screening at one pilot CD4 referral laboratory.

**Methods:**

CD4 test volumes from 2014 were extracted to estimate percentage of CD4 < = 100 cells/μl. Daily average volumes were derived, assuming 12 months per/year and 21.73 working days per/month. Costing analyses were undertaken using Microsoft Excel and Stata with a provider prospective. The cost-per-result was estimated using a bottom-up method, inclusive of test kits and consumables (reagents), laboratory equipment and technical effort costs. The ZAR/$ exchange of 14.696/$1 was used, where applicable. One-way sensitivity analyses on the cost-per-result were conducted for possible error rates (3%– 8%, reductions or increases in reagent costs as well as test volumes (ranging from -60% to +60%).

**Results:**

The pilot CD4 laboratory performed 267000 CD4 tests in 2014; ~ 9.3% (27500) reported CD4< = 100 cells/μl, equivalent to 106 CrAg tests performed daily. A batch of 30-tests could be performed in 1.6 hours, including preparation and analysis time. A cost-per-result of $4.28 was reported, with reagents contributing $3.11 (72.8%), while technical effort and laboratory equipment overheads contributed $1.17 (27.2%) and $0.03 (<1%) respectively. One-way sensitivity analyses including increasing or decreasing test volumes by 60% revealed a cost-per-result range of $3.84 to $6.03.

**Conclusion:**

A cost-per-result of $4.28 was established in a typical CD4 service laboratory to enable local budgetary cost projections and programmatic cost-effectiveness modelling. Varying reagent costs linked to currency exchange and varying test volumes in different levels of service can lead to varying cost-per-test and technical effort to manage workload, with an inverse relationship of higher costs expected at lower volumes of tests.

## Introduction

Cryptococcal meningitis (CM), an opportunistic infection (OI), is a major cause of mortality and morbidity in HIV-positive patients in South Africa due to high local HIV prevalence that ranges between 16.9 and 37.4% across nine provinces (national prevalence of 29.5% is recorded)[1].CM primarily affects patients with a CD4 < = 100 cells/μl [2,3], not yet on antiretroviral therapy (ART) or recently initiated on ART. In South Africa, the prevalence of incident Cryptococcal antigenaemia (CrAg) among patients with a CD4 < = 100 cells/μl is estimated to be between 4 and 7% [4,5].

According to the World Health Organisation (WHO) ‘‘Guidelines for the Management of Diagnosis, Prevention and Management of Cryptococcal Disease’, routine CrAg screening is recommended in ART-naïve adults with a CD4 < = 100 cells/μl and/or where the prevalence of CrAg is high (>3%) [2,6,7]. CrAg positive patients are followed up with pre-emptive anti-fungal therapy to reduce the development of Cryptococcal disease. In line with WHO guidelines [6], the Southern African HIV Clinician’s Society issued local recommendations in 2013 [8] and the national HIV treatment guidelines were revised to include clinician-initiated CrAg screening in 2015 [9]. In South Africa, an alternative screening approach, i.e. reflexed CrAg screening of all routine samples submitted for CD4 testing with counts< = 100 cells/μl in CD4 laboratories, has been proposed as a solution to more efficiently detect early Cryptococcal disease in immuno-suppressed HIV+ patients [2,4]. This approach can reduce CM associated morbidity and mortality of patients identified with early Cryptococcal disease as revealed by recent cost-effectiveness modelling in the context of South Africa and CD4 testing services [10].

In 2012, the National Health Laboratory Service (NHLS) in South Africa in collaboration National Institute of Communicable Diseases (NICD), implemented a pilot study in 8 sites to evaluate reflex CrAg screening on remnant blood from routine CD4 samples with confirmed counts <100 cells/μl[4] using a manual lateral flow assay (LFA) [11](IMMY Mycologics, USA). The NHLS is uniquely placed to implement laboratory based CrAg screening in South Africa through its extensive nationwide integrated tiered CD4 laboratory network of 59 laboratories (at 2014/15) facilitating wide access to services across the country for the staging and/or monitoring of HIV-infected patients [12,13]. During 2014 the NHLS performed 3.9 million CD4 tests, of which 362 000 were reported with a CD4 < = 100 cells/μl [14] (the latter anticipated as the potential annual CrAg screening service load to accommodate a national CrAg screening approach). A tiered CD4 service delivery model (ITSDM) is applied that matches testing demands with appropriate testing capacity/equipment to manage laboratory work load, turn-around-time (TAT) and costs [15]; a CrAg screening program is expected to meet the demands of the busiest sites. The aim of this study was to establish a cost-per-result of reflexed CrAg testing using the LFA assay at one designated pilot testing laboratory designated as a high volume site in keeping with higher tiers of CD4 services. The pilot laboratory was specifically chosen as a high-throughput CD4 testing laboratory proxy for the majority of the CD4 services in South Africa, to establish a base-line cost-per-result that could be extrapolated for preliminary local budgetary planning purposes as well as provide for a basic cost-per-result that underpinned a local cost effectiveness modelling study [10], required prior to proposed national implementation. The study also provides a methodology and a model for the procedures and factors that need to be considered in the course of costing a laboratory test. Consideration is given to costs of reagents and consumables, technical effort and other variables including varying service volumes, error rates and costs of reagents as well as fluctuation of currency exchange, all of which can impact on the final cost-per-result.

## Methods

### Establishing test volumes and workload

CD4 and CrAg test volume data for 2014 (calendar year) from the Tambo-Memorial CD4 pilot laboratory (TM), located in the Ekurhuleni district of Gauteng near Johannesburg, was extracted from the NHLS Corporate data warehouse (CDW). An average daily volume was calculated and used for the costing analysis with 12 months per year and 21.73 working days per month assumed. A 1% error rate was applied to staff and reagent costs.

### Costing method

The costing analysis (S1 Table) was undertaken using Microsoft Excel and Stata. Costs were obtained from NHLS expenditure data as well as manufacturer quotations. A provider prospective was taken, with all costs reported for the NHLS as the provider of reflexed CrAg screening. The main outcome of interest was the cost-per-result. All costs are reported in USD using the prevailing exchange rate of 13.696 ZAR [16]. The Consolidated Health Economic Evaluation Reporting Standards (CHEERS) checklist was used in the preparation of the manuscript [17]. The cost-per-result was estimated using a bottom-up method, inclusive of reagents and consumables, laboratory equipment and staff time.

The cost of organisational infrastructure overheads including capital costs of buildings and laboratory information system as well as logistics and pre-analytical processing are included in the cost of a CD4 result, hence excluded for reflex CrAg screening.

### Reagents

Reagents for the CrAg test consisted of the LFA Cryptococcal kit (including reagents to perform 50 tests), as well as consumables including pipette tips (5–200 μl), gauze, micro-tubes (2ml), latex gloves, and printer toner and paper (to print the LIS worksheet). Quantities of consumables used were assessed during workflow analysis at Tambo Memorial laboratory. The prevailing South African Value added tax (VAT) rate, at 14%, was applied for all reagents and test consumables.

### Laboratory equipment

The LFA is a manual test that does not incur any analyzer costs. Test-specific laboratory equipment costs included a 40μl pipette and specimen rack. Equipment costs were annualized using a 5-year working life and 4% discount rate. VAT was also applied to laboratory equipment.

### Staffing/ technical effort time allocation

Workflow analysis performed during the TM pilot study was used to assess hands-on-time for staff performing CrAg screening. This involved a step-by-step, stopwatch assessment based on a batch size of 30 tests (S2 Table). The attending technologist was required to prepare the LIS work list identifying samples with CD4 < = 100 cells/μl. These LIS identified filed CD4 samples (typically filed in numerical order using the LIS episode number for ease of retrieval) had to be manually retrieved. The technologist then had to label CrAg test tubes and prepare Crag samples in order of the LIS allocated patient work list. Sample preparation included adding diluents and test strips to pre-labelled tubes. Samples were incubated for (10 minutes); individual strip reading followed immediately. For each batch of samples tested a positive and negative control was set up in a similar fashion to patient tests. Results were entered manually onto the printed work list, before being transcribed onto the LIS and reviewed by a senior technologist. Lastly, re-filing of samples according to laboratory standard procedure was done. It was assumed that laboratory staff would not be able to perform other activities while testing CrAg samples. The total time to test a batch of 30 CrAg tests per day was calculated and reported as % full-time equivalent (FTE, based on 8-hour work day with 75 additional minutes for lunch and tea). Mid-point NHLS salary scales for a medical technologist and senior medical technologist were used. The senior medical technologist time was additionally allocated for time taken for authorizing and reviewing of CrAg results.

### Sensitivity analysis

A one-way sensitivity analysis was conducted for a range of error rates, i.e. 3, 5 and 8%. Additionally, a 10 and 20% reduction in the cost of the LFA kit was assessed. To evaluate potential changes due to exchange rate fluctuation, the cost of the LFA kit was increased by 10 and 20%. The sensitivity analysis included the assumption of one full-time technologist (100% FTE of technical effort) at high volume testing facilities. Additionally, sensitivity analysis to establish how daily test volumes would affect cost-per-test outcome was undertaken; volumes of tests were increased and decreased by 10, 20, 30 and 60% to gain insight into how specifically reducing volumes of test performed in any given site would impact on the cost-per-result.

### Statistical analysis

Descriptive statistics were done using STATA to assess median, mean and 95% confidence intervals (95% CI) for cost-per-result calculated for all one-way sensitivity analyses as well as the reported cost-per-result. Additionally, interquartile ranges were reported for the cost per result.

## Results

### Daily CrAg test volumes

The Tambo Memorial laboratory performed 267000 CD4 tests in 2014 of which 27500 reported a CD4 < = 100 cells/μl (9.3%). This equates to an average of 2298 CrAg tests per month. The average number of samples tested per day was106±36.52.

### Workflow analysis and technical effort/ staff time allocation

The workflow analysis revealed that it would take 2 minutes per batch of 30 samples for a technologist to log on to the TrakCare LIS, generate and print the worklist (Table 1 and S2 Table). This process required internet connectivity and a computer; as such, the 2 minutes documented is based on the local bandwidth capacity and LIS terminal availability at the Tambo Memorial laboratory (the TM lab is a clinical pathology lab offering chemistry, haematology, microbiology and CD4 testing in the same laboratory, and is typical of most NHLS laboratory sites). A medical technologist was able to locate ~3 CD4 samples earmarked for CrAg screening per minute from numerically filed CD4 samples. To find an average 106 CrAg samples from typical daily workload of 1,027 CD4 samples per day, at least 10 minutes of technologist time per batch. Doing 3–4 batches per day, equated to ~30–40 minutes of technologist time, per day.

**Table 1:** Number of samples that could be processed per minute by a competent experienced medical technologist for each testing activity required for the lateral flow assay (LFA) assuming a batch size of 30 samples. The total time per activity for a batch of 30 samples are reported as “time per batch”.

Testing Activity	Samples per minute	Time per batch (minutes)
Prepare worklist		2
Find samples	3	10
Prepare 2ml Micro-Tubes	3	10
Add diluent and controls	2	15
Add patient plasma	2	15
Add LFA test strip	7	4.5
10 min incubation		10
Read LFA results, enter worklist	3	10
Enter LFA results on LIS	3	10
File patient samples	3	10
**Total**		**96.5**

The preparation of the 2ml micro-tubes (3 samples per minute) consisted of: (i) placing EDTA samples in the order of the LIS worklist, (ii) labelling each micro-tube with the episode number (for specimen integrity and results tracing) and (iii) matching micro-tubes with patient sample (episode numbers matched). The medical technologist was able to add diluent, patient sample or control sample to micro-tubes at two samples per minute while adding 7 LFA test strips per minute (Table 1).

The 10-minute manufacturer-stipulated incubation period was allocated per batch of 30 CrAg samples. Following incubation, strip results were immediately and consecutively read and results manually entered on the LIS generated worklist (3 samples per minute). Positive samples were verified as positive by a senior technologist.

Medical technologists were able to enter results on the LIS and file samples back in the CD4 racks at a rate of ~3 samples per minute. To enter results on the LIS, staff were required to: (i) log in to the LIS (once off for the batch), (ii) call up the individual laboratory episode number, (iii) enter results after selected the CrAg test method, (iv) save the entered results, and (v) log off LIS. Taking all of the above into account, a laboratory technologist would need to allocate approximately 1.6 hours per batch of 30 CrAg samples per day. At the designated pilot site, TM, this equated to more than 4 hours of technical effort per technologist per day, representing 78% FTE. The impact of staff allocation is further explored in the sensitivity analysis below.

### Reagent and consumable costs

The Cryptococcal LFA kit contributed $2.95 (94.7%) to the reagent costs of $3.11 (Table 2). In comparison, test consumables (Table 2) contributed only $0.16 (5%). The micro tubes required for the LFA assay contributed only 4% to the reagent costs.

**Table 2:** Breakdown of the contribution of the test kit and consumables to the overall reagent cost-per-result.

Test Kit	Cost-per-result	% Total
CrAg LFA Kit (Cryptococcal Antigen LFA)	$2.95	94.7%
**Test Consumables**		
Pipette tips 5-200UL	$0.004	0.1%
Gauze swan	$0.001	0.0%
2ml micro-tubes	$0.124	4.0%
Gloves -latex powder free	$0.003	0.1%
Printer toner	$0.020	0.6%
A4 Paper Bond 80 Grams	$0.000	0.0%
50l Disposal box with lid and liner	$0.008	0.2%
10l Sharps container	$0.005	0.2%
**Total Reagents cost**	**$3.11**	**100%**

### Total cost-per-result

A cost per CrAg result of $4.28 was calculated for Tambo Memorial CD4 laboratory. Overall, reagents contributed $3.11 (72.8%) of the cost-per-result. Based on the initial estimate of 1.6 hours per 30 sample-batch, staff costs contributed~$1.17 (27.2%) to the overall cost-per-result reported here. Minimal laboratory equipment was required, contributing less than 1% ($0.03) to the overall cost-per-result.

### One-way sensitivity analysis

**Fig 1:**
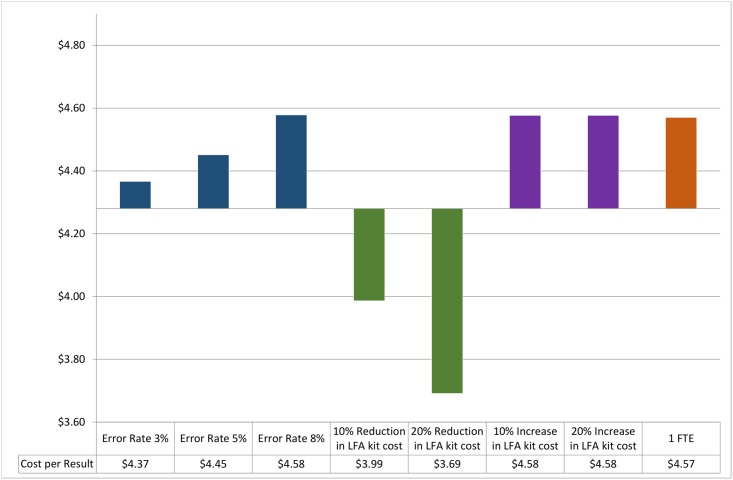
One-way sensitivity analysis of the cost-per-result. One-way sensitivity analysis to assess changes in the cost-per-result based on changes to the error rate, reduction/increase in CrAg LFA reagent cost and allocation of a full-time medical technologist at the Tambo Memorial laboratory.

At a level of processing 106 samples per day, a one-way sensitivity analysis revealed an incremental cost-per-result of $0.08, $0.17 and $0.30 at error rates of 3%, 5% and 8% respectively (Fig 1). A 10% and 20% reduction in the cost of the Cryptococcal LFA resulted in a reduction in the cost-per-result of just $0.30 and $0.59 respectively (Fig 1). Similarly, a 10 or 20% increase in the cost of Cryptococcal LFA kit resulted in an incremental cost of $0.30 and $0.59 respectively (also Fig 1). Finally, increasing staff allocation to one full-time medical technologist resulted in an incremental cost-per-result of $0.29 (this was a practical consideration as only a full time additional staff member could be employed and not a fraction of a staff member) (Table 3). Increasing daily CrAg test volumes resulted in a %FTE range of 55.9–100.7%, confirming the need for an additional full time staff member to manage the workload in a laboratory performing high volumes of CrAg LFA tests (Table 3).

**Table 3:** One-way sensitivity analysis to assess changes in %FTE allocation based on changes in daily CrAg volumes at 10, 20 and 30% increase/decrease.

One-way Sensitivity Analysis	% FTE
+30% test volumes	100.7%
+20% test volumes	92.9%
+10% test volumes	85.8%
**Tambo Memorial (base case)**	**77.9%**
-10% test volumes	70.8%
-20% test volumes	63.0%
-30% test volumes	55.9%

**Fig 2:**
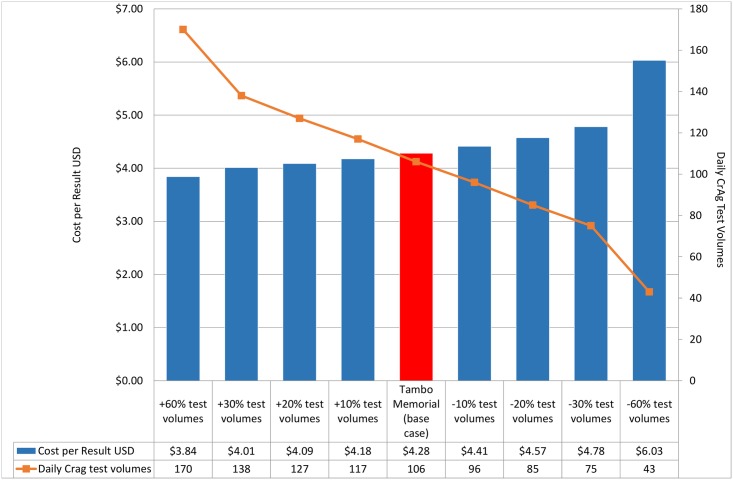
Impact of changes in daily volumes on the cost-per-result. One-way sensitivity analysis to assess changes in the cost-per-result based on changes in daily CrAg volumes at 10, 20, 30 and 60% increase/decrease in relation to base-line cost-per-result.

A mean and median cost-per-result of $4.34 and $4.41 were reported respectively. The 95% confidence interval ranged between $4.18 and $4.51, with an interquartile range of $4.09-$4.5(Fig 2). There was an inverse relationship between volume of tests and cost-per-result outcome. A 10, 20, 30 and 60% increase in daily CrAg volumes reduced the cost-per-result to $4.18, $4.09, $4.01 and $3.84 respectively (Fig 2). Similarly, decreased test volumes at similar increased cost-per-result to $4.41, $4.57, $4.78 and $6.03 at 10, 20, 30 and 60% respectively (Fig 2).

## Discussion

This study has established a calculated baseline cost-per-result for reflexed CrAg testing on routine CD4 samples with counts < than 100 cells/μl, using an LFA assay in the context of a typical busy NHLS CD4 laboratory. Further, this work provides insights into workflow analyses, processes and components required to establish a cost-per-test of reflexed CrAg screening and the related costs. This methodology (S1 Table) can be followed by administrators, policy makers or laboratory managers who aim to gain an understanding and perform such costing exercises in their own environment.

Several assumptions were made in the final cost-per-test calculations including appropriate levels of staff efficiency and competency and adequately filled primary EDTA CD4 samples earmarked for CrAg screening. Prevailing reagent prices at the time of analysis and USD/ZAR exchange rates (ER) (13.696) were used. Careful consideration of exchange rates and its impact on reagent prices is also highlighted here. This is especially important in the context of implementation of a national program, where test volumes can run into several hundred thousand samples per year; cumulatively such perceived small changes/ losses in currency exchange can add substantial costs across a nationwide program, especially where reagents are imported. In South Africa, for example, local exchange rates have varied markedly over the past year, noted to be as high as 14.9030 [18] earlier in 2016. If testing was formally implemented during this period, a reagent increase from $2.92 (13.696 ER) to $3.29 (14.9030 ER) for LFA kits could have been expected, leading to an increased cost-per-result of $4.65. The impact of economy of scale and role of standardised testing and bulk procurement in this context helps to deliver stable national procurement of reagents. In South Africa, large-scale procurement of goods are facilitated through national policy-driven procurement policies in the form of formal open competition for national tenders coupled with fixed-term service level contracts. These regulated processes help to limit price increases and fluctuations linked to currency exchange volatility

This study breaks down a costing analysis into several components to emphasize the aspects that need to be considered when costing a laboratory based test. Reagent costs contributed significantly (72.8%) to the overall cost-per-result (Table 2). The one-way sensitivity analysis looking at a reduction of reagent costs, only slightly reduced the overall cost-per-test; a modest saving of $0.14 and $0.57was revealed with a 10 and 25% reduction in kit costs respectively. Relatively high error rates of up to 8% that could influence the cost-per-result that were also subject to sensitivity analysis but did not markedly alter cost-per-result (ranging from $0.08c to $0.30). Sensitivity analysis was also performed to look at how varying volumes of tests performed in a single site can impact on cost-per-result (Fig 2). An inverse relationship was reported between test volume and cost per result with an almost 50% higher cost-per-test noted when baseline pilot CrAg test volumes fell by 60%. Similarly, when volumes of tests increased by 60%, cost per test reduced to just over $3. This was largely attributed to improved/more effective utilization of staff and reagents at higher test volumes. The CrAg LFA test was developed as a POC test but adapted for laboratory use in the NHLS plot study due to its excellent sensitivity/specificity and ease of use[19]. Costs of producing a reflexed CrAg at the point of care (POC) may be vastly different; this aspect was not included in this work. Therefore, implementation of CrAg screening as a true POC test, i.e. at clinic level, may require adaptation of reported costing assumptions, i.e. including costs related to technical effort, batch size, connectivity and local administration including quality control, stock control, etc. Extrapolating the volume sensitivity analysis (Fig 2) outcome to a context such as a very low volume laboratory or low volume tests expected in a POC setting, suggests that cost-per-result will be considerably higher than $6.

Significant workflow challenges were highlighted during the workflow analysis in the CD4 CrAg screening pilot laboratory. From experience at this pilot site, batches exceeding 30 samples presented challenges for medical technologists who were required to read all results within the allocated 10-minute incubation period. Larger batch sizes also potentially led to transcription errors when manually derived LFA results were entered into the LIS. Limiting the batch size to accommodate manufacturer-required reading times, checking result entry and accommodating varying staff competency and/or efficiency, lead to significantly increased technologist hands-on time, necessitating multiple batches to be performed. Busier testing facilities would require an additional technologist (as additional staff/dedicated CrAg testing technologist) in high-volume sites to accommodate the workload. In the context of testing historical CD4 work load volumes and proportions of samples with CD4 <100 cells/μl, and with ~360 000 tests projected annually, additional potential staffing/ hiring costs outlined above, could have a significant impact on the operational cost of a national screening program.

In South Africa, a relatively resource-constrained country, with a recent history of austerity measures in the NHLS [14], innovative systems that integrate reflexed testing into existing CD4 workflow using existing staffing complement are essential for long term sustainability of the national programs. Laboratories performing in excess of 60 CrAg samples per day would benefit from automated testing platforms. To this end, alternative testing platforms geared for high volumes of CrAg testing have been evaluated [20]. This includes a fully automated enzyme immunoassay (EIA) platform that would increase daily throughput and offer walk-away automation at costs not dissimilar from testing described here. Such systems would potentially decrease hands-on time [21] and the need for a full time staff member to maintain a CrAg reflex service in high volume laboratories. Although EIA platforms come with slightly increased reagent costs, this cost is offset by testing automation, obviating the need for additional FTE/ staffing needs [22]. Additional advantages of EIA platforms include a bi-directional interface that would improve result reporting and prevent transcription errors, as evidenced for LFA testing at the pilot sites.

## Limitations

Overhead costs were excluded, as described under the materials and methods section. Hand-held readers/ electronic strip scanners that may assist with standardising strip reading and semi-automate analysis where LFA testing is used in laboratories or at the POC were not available for inclusion in the pilot study, but may be required for a national roll out.Tests provided for outside of the reflexed model and considered as‘ clinician-initiated’ testing, are expected to include overhead costs of sample collection, pre-analytical processing and logistics and can be expected to be more expensive.

This study was performed at a single busy laboratory and therefore may not be representative of the cost-per-result across laboratories with varying daily volumes. To address this limitation and assess the impact of varying test volumes on cost-per-result, sensitivity analyses were performed looking at an increase but more specifically, where volumes of tests were markedly reduced. Additional work is underway by the authors to establish true costs of a national service across a network of CD4 testing facilities. Innovative automated systems that improve workflow and use the existing NHLS CD4 laboratory staff complement are included.

Cost-effectiveness modelling of the South African National reflexed CrAg screening program has been reported and is not within the scope of this included paper.

## Conclusion

The costing analysis at a busy CD4 laboratory revealed a cost-per-result between $3.69 and $6.03. Further work is underway to assess costs of a national implementation of reflexed CrAg screening.

## Supporting information

S1 Table Costing analyses template. Summary of costing analysis, showing all components used for the calculation of a cost per result.
(XLSX)

S2 Table Workflow analysis. Breakdown of time allocated for all components of testing.
(XLSX)
